# The Role of Bitter-Tasting Substances in Salivation and Swallowing: Results of the Pilot Study

**DOI:** 10.3390/foods14020210

**Published:** 2025-01-11

**Authors:** Ekaterina Oganesiants, Varuzhan Sarkisyan, Anastasiya Bilyalova, Vasily Isakov, Alla Kochetkova

**Affiliations:** Federal Research Centre of Nutrition, Biotechnology and Food Safety, Moscow 109240, Russiaasbilyalova@gmail.com (A.B.); vasily.isakov@gmail.com (V.I.);

**Keywords:** caffeine, vanillin, epigallocatechin gallate, sialometry, surface electromyography, xerostomia

## Abstract

The aim of this study was to investigate the effects of caffeine, vanillin, and epigallocatechin gallate on salivation and swallowing and to find ways to correct their negative effects. Solutions of these substances with an equivalent intensity of bitter taste were compared for this purpose. To compensate for their effect, solutions of adenosine monophosphate, saliva substitute, and their combination were used. The results of the sialometric and surface electromyographic analyses demonstrate that all of the bitter substances studied exert a significant influence on the physiology of salivation and swallowing while exhibiting distinct modes of action. Caffeine has been shown to increase the area under the swallowing electromyographic curve, which is indicative of an increase in maximal amplitude. Epigallocatechin gallate has been linked to a reduction in salivation rate, an increase in duration, and a decrease in maximal intensity of the sEMG curve. Vanillin is demonstrated to reduce the area under the swallowing electromyographic curve due to a decline in both duration and maximal intensity. The addition of adenosine monophosphate to solutions of all substances under study resulted in a convergence of the salivary secretion and swallowing profile toward a profile that is characteristic of water. The findings can be utilized to modify the physiological responses to bitter-tasting substances when developing novel food formulations.

## 1. Introduction

Bioactive compounds are incorporated into food products for the purpose of achieving specific health benefits through the modification of dietary intake. Such substances have the potential to modify the sensory profile of the product, particularly with regard to taste.

Taste perception is a critical factor in the modulation of swallowing function, with distinct tastants influencing both oral preparation and neuromuscular coordination. When tested individually, sour and sweet tastants have been demonstrated to reduce oral bolus preparation time and shorten submental surface electromyography (sEMG) contraction duration in comparison to bitter and salty stimuli [1]. The intensity of a taste stimulus is influenced by a number of factors, including its concentration, taste quality, the age of the participant, and genetic predispositions. These factors have been shown to significantly affect swallowing dynamics. For instance, high concentrations of salty and sour stimuli have been demonstrated to elicit the highest tongue–palate pressures during swallowing [2]. This suggests that specific taste qualities may modulate the force required for swallowing, contributing to a broader understanding of how sensory inputs regulate this reflex.

Of particular note are the impactful effects of bitter-tasting compounds. For example, alkaloids, polyphenols, and glucosinolates, which are found in plants such as coffee, tea, and cruciferous vegetables, are known for their bitterness. Bitter taste is primarily sensed by 25 different TAS2R receptors, a family of G protein-coupled receptors located on the tongue and throughout the gastrointestinal tract. As an illustration of substances with broad application and significance in sensory research within the food industry, caffeine is known to activate the TAS2R7, TAS2R10, TAS2R14, TAS2R43, and TAS2R46 receptors [3]. Vanillin is another such substance; it activates TAS2R14, TAS2R20, and TAS2R39 receptors [4]. Epigallocatechin gallate EGCG activates TAS2R39 and TAS2R43 receptors at micro- and millimolar concentrations [5]. These substances are included in traditional foods and nutraceuticals and together have an effect on one-third of all known human bitter-taste receptors.

It has been shown that particularly bitter tastes have a direct impact on motor control during mastication, extending their influence beyond taste perception [6]. It has been postulated that the prolonged muscle contraction observed in response to bitter stimuli serves as a protective mechanism against aspiration. Furthermore, umami taste may facilitate swallowing, although the evidence remains preliminary and lacks a direct demonstration of its effect on swallowing function [7]. Studies involving healthy adult males have demonstrated that cortical swallowing pathways are influenced by bitter stimuli. These findings indicate a robust interaction between taste perception and swallowing function, which is mediated by the central nervous system [8].

The effects of a bitter taste in foods for special dietary uses affect swallowing, chewing, and salivation, which may be critical for people with various diseases (xerostomia, Sjögren’s syndrome, neurological and muscle disorders). Excluding these substances from the composition of the products may result in a decreased clinical effect of the product itself. Therefore, it is necessary to correct their effects by influencing bitter taste perception (by using bitter taste receptor inhibitors) or by changing the quantity or quality of saliva (by using saliva substitutes).

Several compounds, including sucralose, aspartame, xylitol, sorbitol, cyclodextrins, betaine, malic acid, chlorogenic acid, magnesium sulfate, sodium citrate, sodium acetate, sodium gluconate, and 5′-adenosine monophosphate (AMP), have demonstrated efficacy as bitter taste blockers, thereby facilitating further investigation into the potential for alleviating dry mouth symptoms [9,10,11]. AMP is the safest and most effective on this list. Saliva substitutes (Ss) promote oral moisturization and stimulate saliva secretion [12]. They typically contain ingredients such as xylitol, glycerin, and polysaccharides and minerals [12,13,14,15].

To date, research has not investigated the differences in action of the various bitter taste substances. The influence of a single bitter taste substance (e.g., caffeine or quinine), typically, has been investigated. In addition, there have been no studies on the possibility of correcting their actions.

This study aims to explore the role of three bitter taste substances (caffeine, vanillin, EGCG) in salivation and swallowing. Given the diversity of bitter substances, this study aims to evaluate the differences in the effects of bitter taste substances on salivation or swallowing processes and to identify ways to correct their negative effects.

## 2. Materials and Methods

### 2.1. Materials

Caffeine, citric acid, xylitol and vanillin were obtained from Sigma-Aldrich (St. Louis, MO, USA), EGCG from DSM Nutritional Products Ltd (Basel, Switzerland), and carboxymethyl cellulose from Chongqing Lihong Fine Chemicals (Chongqing, China). Saliva substitute contained citric acid 0.01% (0.005 g per 50 mL), xylitol 2% (1 g per 50 mL), and carboxymethyl cellulose (CMC) (0.05 g per 50 mL). All substances were in sodium-potassium phosphate buffer (0.001 M, pH = 7.1). One molar adenosine monophosphate (AMP) solution (0.036 g per 100 mL) was also used.

### 2.2. Preliminary Sensory Evaluation

The expert panel participants were selected based on their extensive experience in sensory evaluation and their familiarity with bitter taste perception.

All experts participating in the experiment had between 5 and 25 years of experience in the sensory evaluation of food products using various methods. The participants’ ages ranged from 27 to 49 years.

The initial training of experts in the Spectrum^TM^ method was conducted over the course of four sessions. The first of these was an introductory session, during which the evaluation procedure was described. The remaining three sessions were practical in nature and each lasted for one hour. The objective of the experimental sessions was to familiarize experts with the intensity scale, based on control samples exhibiting low, medium, and high intensities of basic tastes and textural characteristics. Following the training period (3 h), experts with an appropriate threshold of sensitivity to the basic tastes were permitted to participate in subsequent Spectrum^TM^ method sessions.

To participate in the experiment, experts who were able to determine the presence of bitter taste in test solutions for the identification of bitter taste (0.195 g/L) were selected according to ISO 3972:2011 [16].

Following the initial training cycle (6 years prior to the current study), experts were reacquainted with the scales of all basic tastes at least twice per year. By the time of the experiment described in this article, experts had accumulated a minimum of 50 h of experience assessing the intensity of various descriptors in foods of different consistencies using the Spectrum^TM^ scales.

Following training, eight experts took part in the study, with the same group involved in each sensory evaluation (3 men and 5 women). The same experts participated in all experiments for each bitter compound. None of the participants reported having any disease, taking medication, or other conditions related to taste disturbances throughout the duration of the experiment.

The Spectrum^TM^ Taste Scales for bitterness intensity were used as a reference, with caffeine serving as a 2-point bitter taste intensity reference. Experts were trained to rate the bitterness of vanillin and EGCG relative to caffeine using an established scale. The experts participated in three one-hour sessions, in which they rated the bitterness of the compounds. Bitter stimuli were presented, and experts rated the bitterness intensity on the Spectrum^TM^ Taste Scales.

Experiments were conducted at least 2 h after last food intake to reduce potential perceptual disruptors. The participants were instructed to refrain from consuming bitter foods, beverages, and cigarettes for several hours prior to the experiment. The participants were advised to drink 300–500 mL of water prior to the experiment.

### 2.3. Sample Evaluation Procedure

Before tasting, experts rinsed their mouths with drinking water, took a few sips of water, waited for 2–3 min and started tasting the samples (Table 1). The duration of each tasting session was 30 min.

The experts took a 5 min break between samples, with a maximum of seven samples per day. During these breaks, experts were advised to drink only water.

The experts took a sample, rinsed it in their mouth for 20 s, and then swallowed it.

### 2.4. Electromyography

Expanding on Neyraud and Peyron’s approach, participants were exposed to different bitter taste stimuli to explore the physiological response [17].

To investigate the impact of bitter substances on salivary secretion, sEMG was used to measure the activity of muscles during swallowing in response to stimulation by bitter compounds.

Swallow recordings were included in the experimental design to gain a comprehensive understanding of the taste perception. Each of the eight experts took part in swallowing recordings while tasting the test substances, with the initial session including drinking water tasting to adapt to the electromyographic recording system and the experimental environment.

During the experiment, the solutions of bitter substances were given to the experts. The experts took a sample, rinsed their mouth with the solution for 20 s, and then swallowed it. The electrical activity of first 2 min after the sip was recorded.

The assessment was conducted using surface electromyography with a Shimmer EMG myograph. The sEMG data were directly captured from the facial skin using standard, non-invasive Ag/AgCl electrodes with a size of 3.2 × 2.5 cm. The entire recording system was operated at a sampling frequency of 1000 Hz. Two pairs of electrodes were attached to the left and right suprahyoid muscles (calculations used the mean between electrodes) with a reference electrode affixed to the dorsal side of the hand. Electrodes coated with a conductive gel were fixed to the cleaned skin over each muscle.

Data were collected using the Shimmer Consensys EMG Kit (Dublin, Ireland), and processing was performed using OriginPro 2015 software using the EMG Toolbar module (Couturier A., EMG Toolbar 5.3, 2018).

To evaluate the maximal amplitude (max), duration and area under the curve (AUC) of the swallowing electromyographic signals after filtration were recorded with high-pass cut-off frequency 5 Hz, min–max normalization and integration.

### 2.5. Sialometric Analysis

Pre-weighed containers with screw-on lids were used to collect saliva. The subjects took samples of substances by rinsing their mouth with one sample for 20 s and then swallowing it. They then used a special container to collect all the secreted saliva for 3 min. The containers were closed with lids to prevent moisture evaporation.

The amount of saliva secreted was determined by measuring the difference between the initial and final container mass.

### 2.6. Statistical Analysis

Statistical analyses were performed using RStudio (2024.4.2.764, R version 4.4.1). Kruskal–Wallis test with Dunn’s test for post hoc multiple comparisons was used to analyze differences between groups. The data is shown as median ± confidence interval. Statistical significance was defined as *p* < 0.05.

## 3. Results and Discussion

### 3.1. Compilation of Bitter Taste Scales

**Figure 1:**
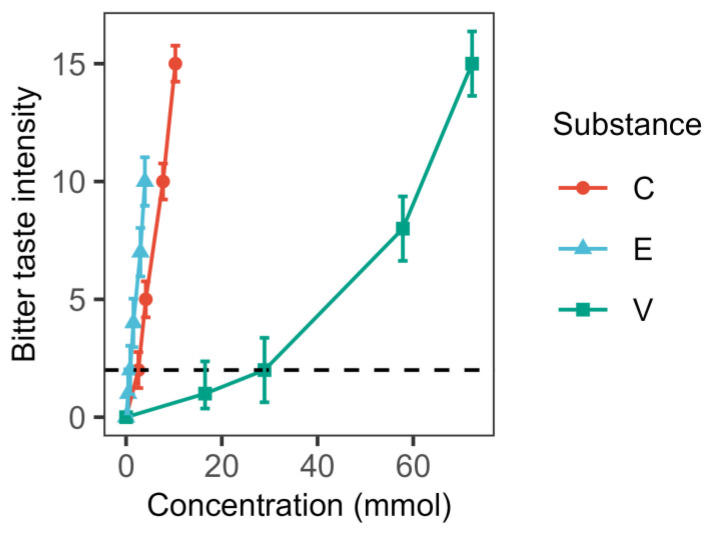
Intensity of bitter taste of substances as a function of concentration. Points indicate means, whiskers—standard deviation (C—caffeine, E—epigallocatechin gallate, V—vanillin).

In the initial phase of the study, a scale for assessing the bitterness of the selected samples of bitter substances (C, V, and E) was developed. The findings of this study are shown in Figure 1.

As shown in the figure, the samples differed in the rate of increase in bitter taste depending on the concentration. The lowest rate was observed for the vanillin solution (V). The weak perception of bitter taste for vanillin solutions began at a concentration of 15 mmol, which is consistent with the perception of the concentrations required to activate the bitter taste receptors TAS2R14, TAS2R20, and TAS2R39 [4]. The nonlinear increase in the intensity of the bitter taste of vanillin with increasing concentration was observed at the beginning of a sharp increase stage at concentrations above 28 mmol. It should be noted that the bitter taste perception of vanillin is complicated by its ability to activate vanilloid receptors, which may cause burning sensations in the mouth and thereby influence the evaluation of bitterness.

Epigallocatechin gallate had the highest rate of concentration-dependent increase in bitter taste intensity in the concentration range studied. Our findings are in agreement with previously published data [18]. EGCG (E) has an astringent taste (astringency), in addition to bitterness, which can also influence the perception of bitter taste. The manifestation of this descriptor is due to the binding of EGCG to salivary proteins, reducing its protective function in the oral cavity (article reference), which may lead to a more astringent perception of bitter taste.

The data obtained for caffeine (C) were expected to be consistent with the Spectrum^TM^ Taste Scales [19]. Caffeine had no extraneous flavors interfering for bitterness determination.

Based on the study, it was concluded that the C, E, V solutions were characterized by 2 points of bitter taste at concentrations of 2.57 mmol; 0.87 mmol; 28.92 mmol, respectively. These concentrations were used in subsequent studies. The concentrations selected for this study are within the normal ranges for traditional food products. The caffeine content of coffee drinks is known to vary depending on numerous factors, with an average content of 1 g/L being reported in the existing literature. In this experiment, the caffeine content was found to be 0.5 g/L [20]. The EGCG content of brewed green tea has been documented as 0.5 g/L [21], and this value was 0.4 g/L in this experiment. The concentration range for vanillin in food products is reported to vary from 0.32% to 1.44% for yogurt, for example [22]. The vanillin solution concentration in the present study was 4.4 g/L, which is equivalent to 0.44%.

The samples described in Table 1 with bitter concentrations as selected above from the study were used for further analysis.

### 3.2. Evaluation of Bitter Taste

**Figure 2:**
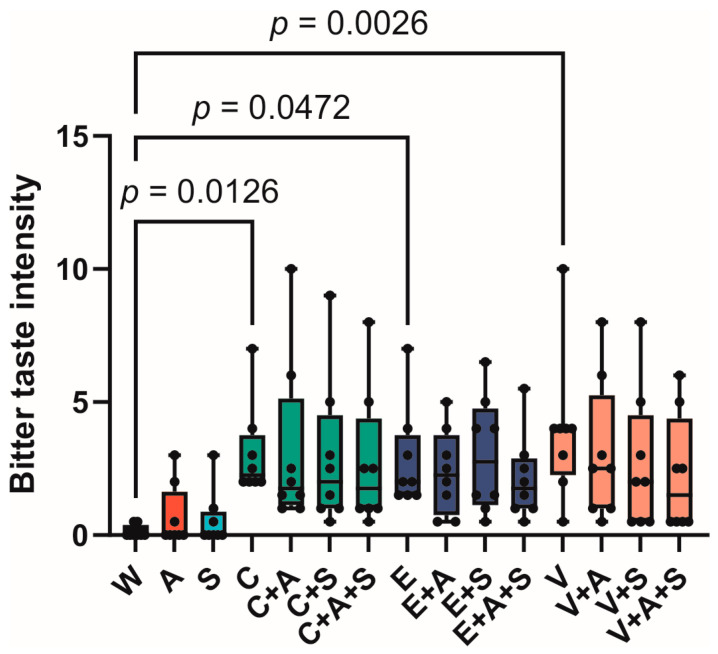
Intensity of bitter taste of the tested samples. Boxes represent confidence intervals, whiskers—minimal and maximal values (A—AMP, C—caffeine, E—epigallocatechin gallate, S—saliva substitute, V—vanillin, W—water).

An organoleptic evaluation of the samples was carried out according to the methodology described in the Materials and Methods section, the results of which are presented in Figure 2.

As anticipated, the median values for bitter taste intensity of the studied solutions fell within the range of two points (2.00 ± 0.01). The intensity of the bitter taste in water was found to be 0.00 ± 0.01. A slight increase in the intensity of the bitter taste was observed in solutions A (0.00 ± 0.03) and S (0.00 ± 0.02), which may indicate the presence of taste characteristics that can be interpreted under experimental conditions as bitter.

The statistical analysis revealed that the initial bitter solutions, designated as C, E, and V, exhibited statistically significant differences in the intensity of the bitter taste when compared to water. The median intensity of the bitter taste for these solutions was 2.25 ± 0.04 (*p* = 0.0126) for solution C, 2.00 ± 0.04 (*p* = 0.04) for solution E, and 4.00 ± 0.06 (*p* = 0.003) for solution V. The increased intensity of the bitter taste sensation caused by vanillin may be attributed to its burning effect.

### 3.3. Sialometry

**Figure 3:**
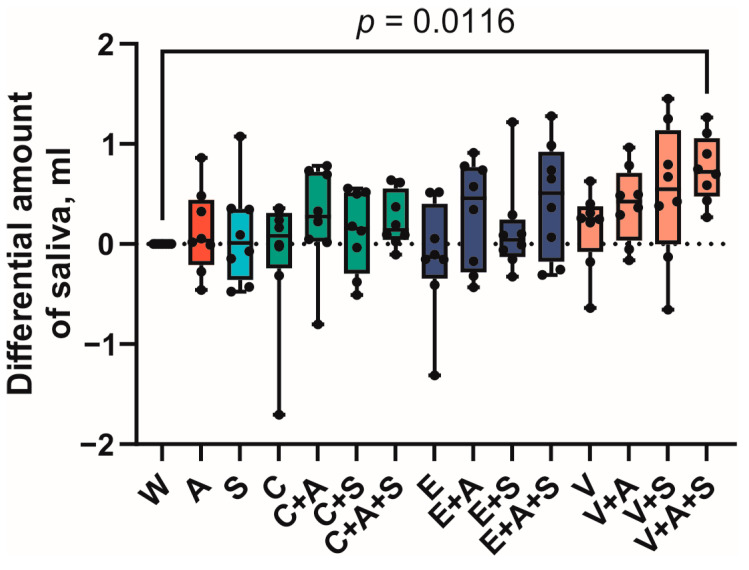
Differential amount of the secreted saliva. Boxes represent 95% confidence intervals, whiskers—minimal and maximal values (A—AMP, C—caffeine, E—epigallocatechin gallate, S—saliva substitute, V—vanillin, W—water).

The results of the sialometric study are shown in Figure 3. Given that the studies were conducted over several days, the data are presented in the form of the difference in the mass of saliva secreted after ingestion of the respective samples and the mass of saliva secreted after ingestion of water (on that day) as a control.

As illustrated in Figure 3, the mass of saliva secreted following the ingestion of the V + A + S sample (0.7229 g ± 0.0074 g) exhibited a statistically significant difference (*p* = 0.0116) from the mass of saliva secreted following the ingestion of water. Additionally, the majority of vanillin samples exhibited a marked tendency to stimulate salivary secretion. The lowest value of relative mass of secreted saliva was observed in sample E, with a recorded value of −0.1298 g ± 0.0128 g.

### 3.4. Electromyographic Studies

**Figure 4:**
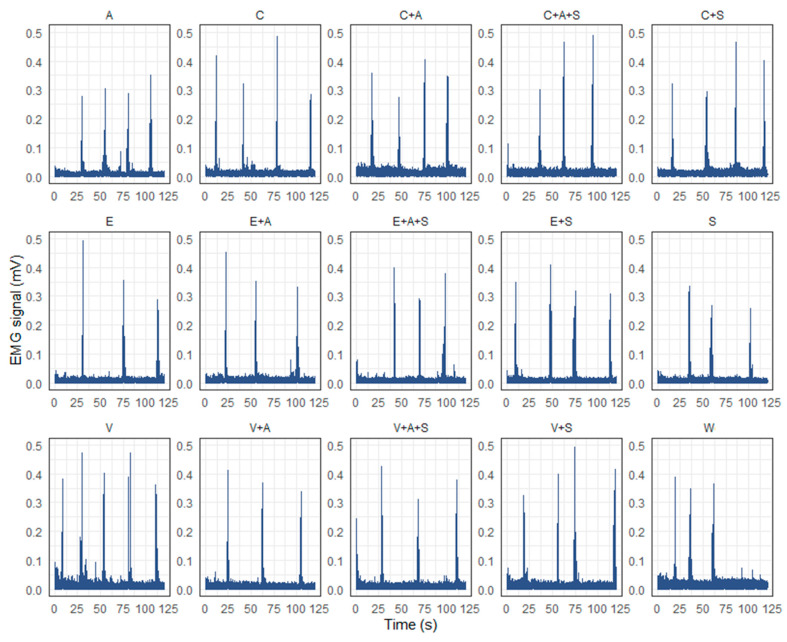
Characteristic EMG curves for each sample obtained from a single expert after filtration, normalization and signal integration. (A—AMP, C—caffeine, E—epigallocatechin gallate, S—saliva substitute, V—vanillin, W—water).

Characteristic EMG curves for each sample obtained from a single expert are shown in Figure 4. As illustrated in the figure, there are notable discrepancies in the number and frequency of sips taken by the examiner during the two-minute measurement period, as well as the time of the initial sip onset. However, a statistical analysis of these indicators based on the results of sEMG studies of all experts revealed that there were no significant differences between the samples (*p* > 0.05). Accordingly, these indicators were excluded from subsequent evaluation.

In some cases, the number of sips recorded by experts was limited to two. Consequently, all studies were conducted exclusively for the initial two sips for all experts across all samples, ensuring the integrity of the statistical analysis. Additionally, no notable discrepancies were identified between the attributes of the first and second sips under examination.

**Figure 5:**
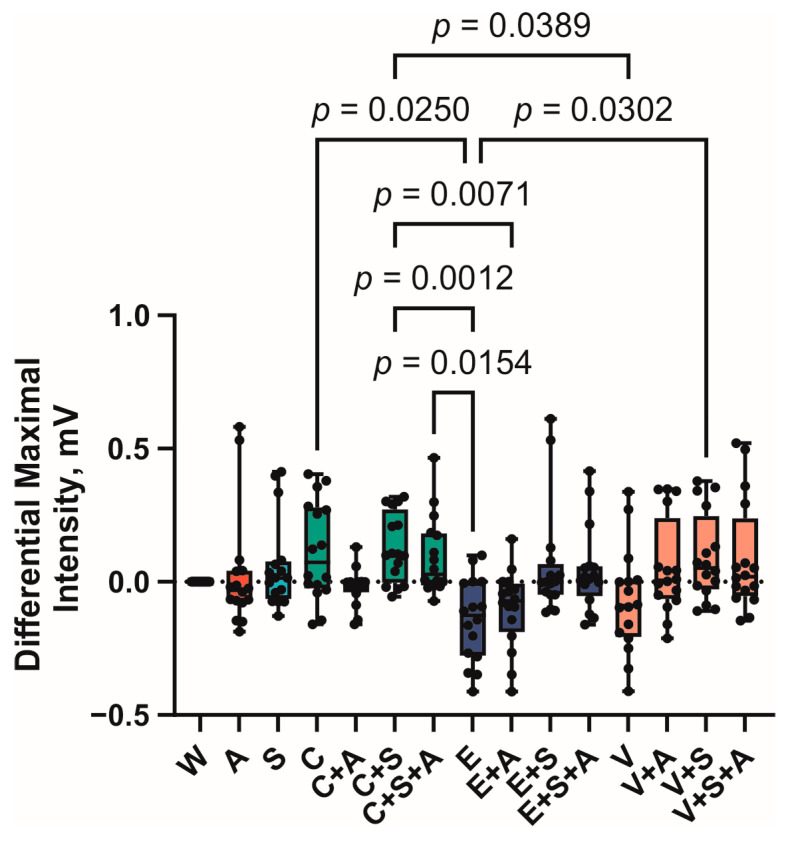
Differential maximal intensity of sEMG curve. Boxes represent 95% confidence intervals, whiskers—minimal and maximal values (A—AMP, C—caffeine, E—epigallocatechin gallate, S—saliva substitute, V—vanillin, W—water).

The results revealed statistically significant differences between the characteristics of the sEMG curves for the measures of maximum amplitude, sip duration, and area under the curve. No statistically significant differences were found between the maximal amplitude values of bitter-tasting solutions and water (Figure 5).

A notable (*p* = 0.025) discrepancy was observed between the differential maximum sEMG signal intensity at swallowing for samples C (0.1 mV ± 0.5 mV) and E (−0.2 mV ± 0.5 mV). Sample V also exhibited a decline in this parameter (−0.1 ± 0.6 mV); however, the differences from sample C were not statistically significant. The sEMG signal intensity increased in samples containing caffeine and vanillin when both AMP or saliva substitute or a combination of both were added.

**Figure 6:**
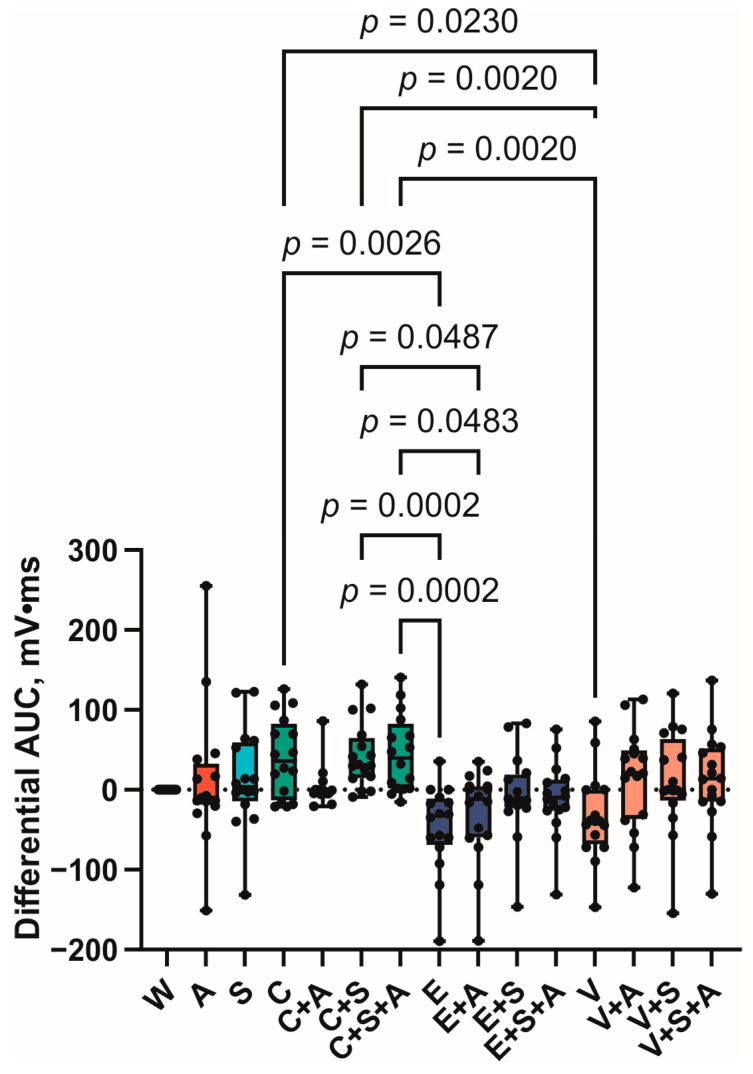
Differential area under the curve of EMG swallowing curve. Boxes represent 95% confidence intervals, whiskers—minimal and maximal values. (A—AMP, C—caffeine, E—epigallocatechin gallate, S—saliva substitute, V—vanillin, W—water).

Similar patterns were observed in the variation of the area under the sEMG curve (Figure 6). A significant difference (*p* = 0.0026) was observed between the AUC for samples C (50 mV ± 10 mV*ms) and E (−20 mV ± 15 mV). As with the previous indicator sample, sample V also exhibited a decrease, with a value of −20 mV ± 10 mV. However, in this case, the differences from the sample were found to be statistically significant (*p* = 0.023).

**Figure 7:**
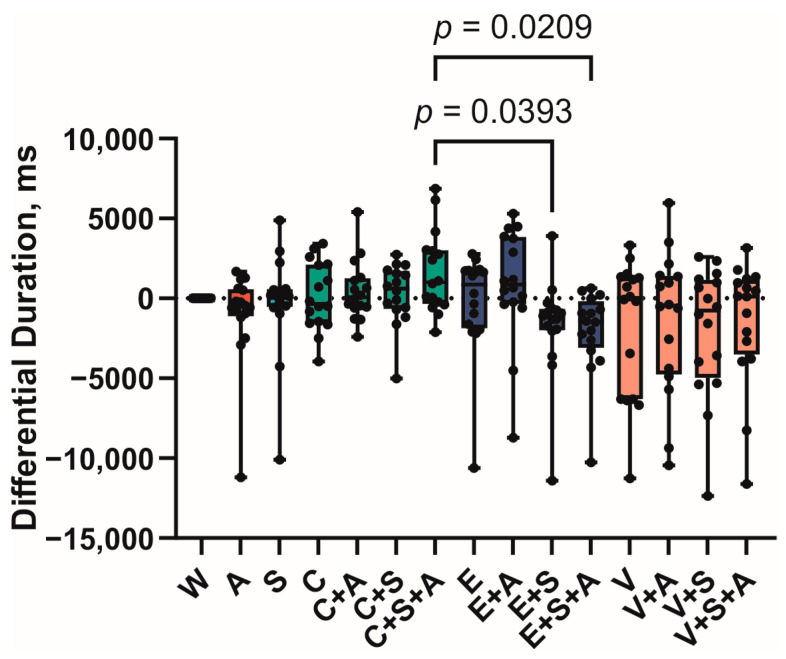
Differences in the duration of sip. Boxes represent 95% confidence intervals, whiskers—minimal and maximal values. (A—AMP, C—caffeine, E—epigallocatechin gallate, S—saliva substitute, V—vanillin, W—water).

The variation in the sip duration value was less pronounced (Figure 7). Two significant differences were observed between the groups C + S + A and E + S + A (*p* = 0.0209), and C + S + A and E + S (*p* = 0.0393). These findings suggest that the effect of saliva substitutes on swallowing and salivary secretion processes may differ in the presence of different bitter-tasting substances.

The large variation in the values obtained by sEMG compared to sensory analysis and sialometry may be attributed to several factors. Primarily, the recorded potential is the summation of the action potentials from numerous muscle fibers. The duration, amplitude, and shape of the action potential are dependent on the number of individual muscle fibers that constitute the muscle. As these characteristics are subject to inter-expert variability, this may contribute to the observed variation in the characteristics of the sEMG curve [23,24,25].

Principal component analysis was conducted to enable a comprehensive evaluation of the complex physiological responses to caffeine, vanillin, and epigallocatechin gallate solutions, as well as the impact of AMP and saliva substitute.

**Figure 8:**
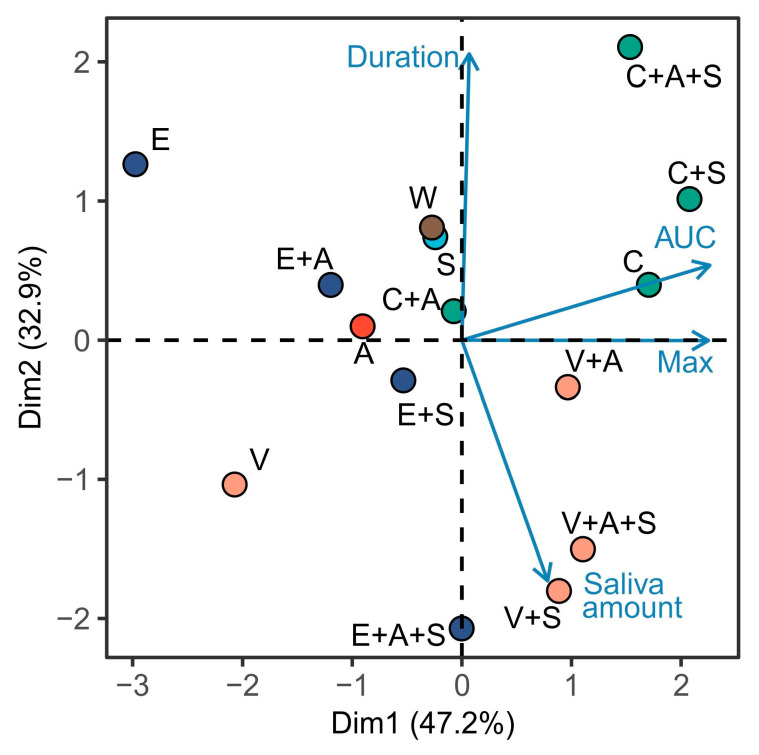
Results of analysis of the obtained data using principal component methods (A—AMP, C-caffeine, E—epigallocatechin gallate, S—saliva substitute, V—vanillin, W—water control).

The application of the principal component analysis method enabled the identification of the primary regularities of changes in the processes of salivation and swallowing under the influence of the studied stimuli (Figure 8).

Using the differential measures of secreted saliva amount along with the sip duration, maximum intensity and AUC of the sEMG curve, it was possible to describe 80.1% of the variation between samples. The first dimension describes 47.2% of the variation and is characterized predominantly by the values of the AUC and maximal signal intensity (positive correlation). The second dimension described 32.9% of the variation between samples and was characterized predominantly by the values of sip duration (positive correlation) and saliva amount (negative correlation).

The results of the principal component analysis clearly demonstrate that the studied bitter-tasting substances exhibit distinct modes of action. Caffeine has been shown to increase the area under the swallowing electromyographic curve, which is indicative of an increase in maximal amplitude. Epigallocatechin gallate has been linked to a reduction in salivation rate, an increase in duration, and a decrease in the maximal intensity of the sEMG curve. Vanillin is demonstrated to reduce the area under the swallowing electromyographic curve due to a decline in both duration and maximal amplitude.

Adding AMP to solutions of all bitter substances led to a significant convergence of the samples (C + A, E + A, V + A) toward water (W) on the coordinate plane. This suggests that AMP is effective in mitigating the adverse reaction to bitter substances, even at concentrations where the bitter taste is partially retained.

The effect of AMP may be due to its ability to block or mask the bitter taste. AMP exerts its effect through the inhibition of peripheral taste. The glossopharyngeal nerve innervates taste receptor cells in the tongue and is responsive to bitter stimuli [10]. It is shown that the application of 0.1 mM AMP resulted in a notable inhibition of the nerve responses to bitter compounds, including quinine and denatonium benzoate. It is hypothesized that AMP may alter the G-protein receptor coupling [26]. Furthermore, the bitter taste of caffeine may also be attributed to its capacity to inhibit the adenosine receptor (A2B and A1), of which adenosine monophosphate serves as a specific agonist [27]. In this regard, AMP may also exert a higher beneficial influence on the swallowing and salivation processes affected by caffeine indirectly, rather than through TAS2R receptors.

The difference in the nature of EGCG action compared to caffeine may be due to the local irritant action caused by binding with saliva proteins, which have an astringent action [28]. Vanillin activates the vanilloid receptor TRPV1. Stimulation of the TRPV1 receptor by vanillin produces a burning or warm sensation similar to the action of capsaicin, although to a lesser extent.

In this regard, it can be assumed that the astringent and warm sensation of EGCG and vanillin is more significant in the processes of salivation and swallowing; therefore, the addition of a salivary substitute might be beneficial to reduce the irritant action. The saliva substitute used in this study was a mixture of components with different effects. Xylitol, citric acid, and CMC are used as salivary substitutes to enhance salivation and flavor perception by reducing the intensity of bitterness. Xylitol is a polyol that stimulates salivation, helps improve flavor perception, and reduces dry mouth. The increased salivation caused by xylitol may help dilute bitter substances in the mouth, thereby reducing their perception. Xylitol does not interact directly with TAS2R or TRPV1 receptors, but its sweetness and cooling effect may mask the bitter taste through interaction with TRPM8 (menthol receptor) [29]. Citric acid increases salivation and is often used to improve the flavor of foods. It does not directly affect TAS2R or TRPV1 receptors, but increased salivation may reduce the perception of bitterness by diluting bitter compounds. The main role of CMC in saliva substitutes is to create the necessary consistency (thickener and stabilizer). Thus, CMC can facilitate swallowing. It does not interact directly with TAS2R or TRPV1 receptors but can influence taste perception by changing the texture and coating the oral and oropharyngeal mucosa.

Nevertheless, the findings of the present study indicate that the use of salivary fluid substitute, especially in combination with AMP in EGCG, caffeine, or vanillin solutions, had a negative impact on salivary secretion and swallowing processes. This was evidenced by the greater distance of the salivary fluid substitute samples from the water sample, with the salivary fluid substitute sample exhibiting the greatest similarity to water on a PCA plane.

A limitation of this pilot study is the relatively small sample size of the experts who took part in the experiments. Further studies with a larger number of participants are needed to confirm the findings. Additionally, it may be advisable to optimize the composition of the salivary fluid substitute to correct the physiological response in the presence of bitter substances.

## 4. Conclusions

It was shown that all the bitter-tasting substances studied affected salivary secretion and swallowing processes.

The results of the sialometric and surface electromyographic analyses demonstrate that all of the bitter substances studied exert a significant influence on the physiology of salivation and swallowing, while exhibiting distinct modes of action. The administration of a caffeinated solution was demonstrated to increase the area under the swallowing electromyographic curve, which is suggestive of an increase in the maximal amplitude of the electromyographic waveform. Epigallocatechin gallate has been linked to a reduction in salivation rate, an increase in duration, and a decrease in the maximal amplitude of a sip. Vanillin, meanwhile, has been demonstrated to reduce the area under the swallowing electromyographic curve as a result of a decline in both the duration and maximal amplitude. Furthermore, the addition of adenosine monophosphate to solutions of all substances under investigation resulted in a convergence of the salivary secretion and swallowing profiles towards a profile that is characteristic of water.

A broad interpretation of the results is constrained by two factors. Firstly, the expert panel was limited in size, which can often result in a lack of statistical power. Secondly, the participants were not randomized by factors that determine selectivity to individual bitter taste substances, such as genetics, age, or medical limitations. Despite these limitations, the results provide a compelling rationale for the implementation of further targeted studies of bitter-tasting substances. These studies should focus on specific groups of individuals, including children, the elderly, individuals undergoing chemotherapy, radiation therapy, or those suffering from Sjögren’s syndrome. This is particularly relevant when considering the incorporation of bitter-tasting substances into new types of food products.

## Figures and Tables

**Table 1:** Composition of tested samples.

**Sample Name**	**Volume, mL**					
	C	V	E	S	A	W
W	-	-	-	-	-	30
C	15	-	-	-	-	15
C + S	15	-	-	15	-	-
C + A	15	-	-	-	15	-
C + S + A	15	-	-	7.5	7.5	-
V	-	15	-	-	-	15
V + S	-	15	-	15	-	-
V + A	-	15	-	-	15	-
V + S + A	-	15	-	7.5	7.5	-
E	-	-	15	-	-	15
E + S	-	-	15	15	-	-
E + A	-	-	15	-	15	-
E + S + A	-	-	15	7.5	7.5	-
S	-	-	-	15	-	15
A	-	-	-	-	15	15
S + A	-	-	-	15	15	-

(A—AMP, C—caffeine, E—epigallocatechin gallate, S—saliva substitute, V—vanillin, W—water).

## Data Availability

The original contributions presented in the study are included in the article. Further inquiries can be directed to the corresponding author.
